# Decision-Making Processes in the Workplace: How Exhaustion, Lack of Resources and Job Demands Impair Them and Affect Performance

**DOI:** 10.3389/fpsyg.2017.00313

**Published:** 2017-05-05

**Authors:** Andrea Ceschi, Evangelia Demerouti, Riccardo Sartori, Joshua Weller

**Affiliations:** ^1^Human Sciences, University of VeronaVerona, Italy; ^2^Human Performance Management, Industrial Engineering and Innovation Sciences, Eindhoven University of TechnologyEindhoven, Netherlands; ^3^Developmental Psychology, Tilburg UniversityTilburg, Netherlands

**Keywords:** decision-making competency, decision environment management, self-regulation, job demands, job resources, exhaustion

## Abstract

The present study aims to connect more the I/O and the decision-making psychological domains, by showing how some common components across jobs interfere with decision-making and affecting performance. Two distinct constructs that can contribute to positive workplace performance have been considered: decision-making competency (DMCy) and decision environment management (DEM). Both factors are presumed to involve self-regulatory mechanisms connected to decision processes by influencing performance in relation to work environment conditions. In the framework of the job demands-resources (JD-R) model, the present study tested how such components as job demands, job resources and exhaustion can moderate decision-making processes and performance, where high resources are advantageous for decision-making processes and performance at work, while the same effect happens with low job demands and/or low exhaustion. In line with the formulated hypotheses, results confirm the relations between both the decision-making competences, performance (i.e., in-role and extra-role) and moderators considered. In particular, employees with low levels of DMCy show to be more sensitive to job demands toward in-role performance, whereas high DEM levels increase the sensitivity of employees toward job resources and exhaustion in relation to extra-role performance. These findings indicate that decision-making processes, as well as work environment conditions, are jointly related to employee functioning.

## Introduction

In complex social environments such as workplace organizations, decision-making has been considered an important factor (along with commitment, work engagement, etc.) contributing to organizational efficiency and workplace satisfaction. For this reason, such approaches as the model of organizational choice (Cohen et al., 1972), strategic decision-making (Eisenhardt and Zbaracki, 1992) and naturalistic decision-making (Pliske and Klein, 2003) have been formulated based on the investigation of the role of the organizations on decision makers. Indeed, classical studies in the decision-making domain usually have focused on the internal validity of the research and are therefore frequently carried out in experimental settings. The focus of organizational studies is mostly based on psychometric instruments exploring individual differences by using within-person studies, while decision-making research makes extensive use of experiments (Dalal et al., 2010). However, research has been gaining increasing appreciation for individual differences in decision-making processes and decision-making styles, the antecedent factors that may predict sound decision-making, and the predictive validity of rational responding (e.g., Parker and Fischhoff, 2005; Bruine de Bruin et al., 2007; Appelt et al., 2011; Weller and Tikir, 2011; Weller et al., 2015). For instance, Miller and Byrnes (2001) developed a self-report measure to assess individual differences in “*decision-making competency*,” which can be characterized as the tendency to be self-regulated and use metacognitive processes to examine choice options and master decisions. This definition has been supported by studies involving objective measures of decision-making competence^1^ (Parker and Fischhoff, 2005; Bruine de Bruin et al., 2007; Stanovich et al., 2008).

Although prior work has supported the notion that individual differences in decision-making competency (DMCy) exist and possess considerable predictive validity (e.g., Miller and Byrnes, 2001; Bruine de Bruin, 2005; Parker and Fischhoff, 2005), no empirical research, to our knowledge, has directly explored its associations with workplace and psychological variables such as job performance, job demands, job resources, exhaustion, etc. This contribution presents a bridge between research advances in decision-making and I/O research, in relation of variables of one of the most comprehensive psychological model: the JD-R one (Demerouti et al., 2001). The aim is to enlighten the role of such decision-making differences and some organizational (i.e., job demands, job resources) and psychological variables (i.e., exhaustion) which can positively or negatively affect performance. For instance, studying the relationships between the DMCy and variables abovementioned, provide new insights about how the work environment and connected psychological variables interfere with decision-making processes (e.g., is the presence of job resources helpful for the decision maker? Do low job demands link to better quality of choices made at work? How can exhaustion alter decision-making?). The role of work environment and its management are considered in this study as a key skill, namely decision environment management (DEM), defined as the sensitivity to the work environment capable of having an effect on complex decision-making processes (Wood and Bandura, 1989). In relation to the aim of this study, these two decisional competences (i.e., DMCy; DEM) are supposed to be related to performance and moderated by components enclosed in the JD-R model.

### Decision-making Competency as a Predictor of Task Accomplishment at Work

Evidence of the predictive validity has showed that lower decision-competence is related to greater risk-taking and potentially maladaptive behaviors (Parker and Fischhoff, 2005; Del Missier et al., 2011; Weller et al., 2012, 2015). If decision-making competence reflects the tendency to approach decisions from a perspective that stresses quality of the decision process rather than solely focusing on immediate outcomes, one might expect that greater decision-making competence also will be associated with lower incidence of behaviors that may bear adverse long-term consequences. For instance, being reflective and gathering enough information before making decisions, relying on sense of self-determination when critically evaluating options and being mindful in relation to choice consequences, all these skills should be able to address toward long-term outcomes. Such components have been selected by Miller and Byrnes (2001) as fundaments of the DMCy conceptualization which relies on the *“self-regulation model of decision-making”* in which a self-regulated decision maker is an individual who sets adaptive targets and takes proper measures to achieve such tasks (Byrnes et al., 1999; Byrnes, 2013).

In complex environments such as organizations, a competent decision-maker requires a variety of cognitive skills to continuously search for information to improve work performance (Bandura and Jourden, 1991). This process involves developing, comparing, and mastering choices while at the same time dealing with cognitive limitations, heuristics and biases and individual inclinations that can impair the accomplishment of task targets (Byrnes et al., 1999). In this sense, a higher level of DMCy could represent an explicative antecedent of task accomplishment at work. This is in accordance with the self-regulation theory, where people cannot successfully adapt to the work environment until they develop a sense of control over behavioral processes (Zimmerman and Bandura, 1994; Schunk and Zimmerman, 2003). These adjustments stimulate the development of strategies to overpower decision-making deficiencies given by harsh work conditions (Miller and Byrnes, 2001).

### Decision-making Impairment Due to Exhaustion

The study of the regulation processes in relation to decisions has been addressed in the organizational domain because it has been recognized to be at the root of many problems of underachievement at work (Lord et al., 2010). In some professions, such as the medicine, law, and finance, fatigue due to an excessive number of choices can impair the self-regulation mechanisms (Vohs et al., 2005; Baumeister and Vohs, 2007; Demerouti and Bakker, 2008). The effort required in decision-making processes rapidly depletes personal resources, thus leaving the executive function less efficient when performing other tasks. As information processing increases, greater cognitive resources are required for a competent functioning (Wood et al., 1990). When individuals reach the limits of the cognitive capabilities, performance can be undermined because attention is diverted to self-evaluative concerns about the consequences of failure (Humphreys and Revelle, 1984). Considering the individual differences studies reported above, this effect could be more pronounced in less competent self-regulated decision-makers. At support of this speculation, decades of I/O psychology studies have widely analyzed performance impairment due to exhaustion as a consequence of intense physical, affective, and cognitive strain (Demerouti et al., 2001; Brotheridge and Grandey, 2002; Goldberg and Grandey, 2007; Deligkaris et al., 2014). Literature has deeply documented how the exhaustion induced by depletion of energy can long-term decrease performance, and how individuals use performance-protection strategies (Bakker et al., 2004; Schaufeli and Taris, 2014). The more the cognitive activation and/or effort at work and the more the physiological costs for the individual are demanding, performance protection is achieved by means of active control of cognitive information processing (Robert and Hockey, 1997). The long-term effects of such process may be emptying the individual’s stamina and personal resources, resulting in a burnout condition and ultimately affecting performance (Demerouti et al., 2001).

Aside from psychophysical individual strategies, organizations can deal with employee’s exhaustion by leveraging job resources, which are work aspects important for goal achievement, personal growth and for minimizing labor costs, such as: supervisor feedbacks, opportunity for development, social support and rewards, etc. (Bakker et al., 2003). Developing decision rules for an optimal adjustment level of instructive feedback to goal attainments or the use of social rewards revealed to have a positive effect on work outcomes (Wood et al., 1990), in particular on extra-role performance (i.e., behaviors that support the environment in which task are performed; Demerouti et al., 2015). Similarly, Bakker et al. (2004) found job resources (e.g., increasing work autonomy and social support) to be the strongest predictors of extra-role performance, whereas the absence of them can negatively affect engagement and performance too. Organizational resources have a different impact on employees’ performance, depending on the subject’s attitudes and on perception of utility that individuals have of them (e.g., some employees could find in social support a valuable job resource, for others autonomy is more important than feedbacks from the supervisor, etc.). To maintain satisfactory performance levels, organizations have to master a complex set of decision rules on how to best match employee attributes and sensitivity toward the resources implemented (Wood et al., 1990).

### The Work Environment Role for a Comprehensive Understanding and Mastering of Decision-making Processes

Until now, the DMCy has been the only decision-making construct here provided to explain the mastering of self-regulation processes in setting choices and achieving targets. Explicability limits related to such a construct belong to the paradigm of decision-making research, traditionally based on laboratory studies and focused on the internal validity of such constructs. In general, these decision-making approaches adapted to workplace studies lack an adequate consideration of the work environment role, especially in relation to the effects of the last economic crisis, the business downsizing and consequently higher job demands for leftovers, increasing job insecurity and uncertain professional paths (Ceschi et al., 2016). Organizational jobs that traditionally occurred within a single context are nowadays replaced by boundaryless, self-managed individual work stories, where people are constantly asked to shift roles, enhance capabilities, and re-adapt to new work environments (Leana and Barry, 2000). About the individual, it means that new psychological issues related to uncertain professional paths give evidence of new patterns of outcomes, such as a lack of motivation at work, low job performance and more burnout incidence. On the other side, in order to cope with that, HR are introducing new organizational models and interventions to foster workplace adaptation (e.g., empowerment policies, participative decision-making, innovation workplace interventions, job crafting) (see Men and Stacks, 2013; Sartori et al., 2013; Demerouti and Bakker, 2014).

In light of these recent consequences, work environment has assumed more and more relevance as determinant of the decision processes made at work by every member of the organization. The effects of environmental factors on the decision-making processes assume particular importance in relation to the organizational aims. As seen, feedback and social rewards can positively affect employee’s co-working, but the impact of these variables is intrinsically related to the sensitivity toward them. In this context, participative decision-making is a good example of how some individuals, inclined to use a collaborative work approach, can perform well in presence of a positive environment (Lowin, 1968). For example, good relations with collaborators promote a participative decision-making style, which in turn can elicit prosocial behavior at work (i.e., extra-role performance; De Dreu and Weingart, 2003; Kozlowski and Ilgen, 2006; Ceschi et al., 2014). Instead, negative environmental variables, such as career breakdowns, lack of job opportunities, absence of colleague support can weaken the efficiency of decision-making processes by impacting the self-regulatory mechanisms that regulate enthusiasm and accomplishment satisfaction (Wood and Bandura, 1989; Wood et al., 1990; Bandura, 1997). In such settings, self-regulatory mechanisms may be hindered by the lack of such resources, resulting in lower performance and motivation, especially in relation to the prosocial behavior in the workplace (Baruch et al., 2004).

With these points in mind, we can state that the capacity to manage negative environmental conditions and take advantage of positive environments is nowadays an essential workplace competence for consistency in decision-making and, ultimately, for better performance. It is a skill intrinsically related to awareness, intrinsic management, and self-regulation in relation to organizational variables, rather than actual decisions at hand (Wood et al., 1990). Although the effective management of environmental conditions can provide a clearer space in which decisions may be made, these should be considered distinct concepts. We referred to this composite skill as decisional environment management (DEM), firstly conceptualized by Bandura and Jourden (1991) as operational causal structures examined within context of managerial decision-making in dynamic environments. Their research position was directed to examine decisions in dynamic organizations while the individual is coping with ongoing activities, because already at the time: *“much of the research on human decision-making examines discrete judgments in static environments under no taxing conditions”* (p. 941). Such interactional causal structures were defined through a triadic model of the antecedes of the mastery of the decisional environment, namely: the cognitive determinants and the quality of analytic thinking, the behavioral mechanisms of the choice management and the properties of the organizational environment. Applied results of such a model confirmed that several factors might account for differential impact on decision processes, in which cognitive regulatory mechanisms firstly alter the systematic exploratory strategies, and the organizational management at second stage (Cervone et al., 1991).

Because of the primary importance given to the decision environment model regarding managerial decisions (like most of the research of the last century has focused on decision-making applied to organizations), a revised version of DEM with the intent of extending this model to different job categories, even subordinates, has introduced the role of colleagues and of supervisor and as interpersonal behavioral determinants of choice in the workplace (Ceschi et al., submitted). Considering the renewed role of work environment on decision-making due to unsure professional pathways, with the emerging of new boundaryless organizational structures and empowerment policies (Men and Stacks, 2013; Cummings and Worley, 2014; Laschinger et al., 2014; Di Fabio et al., 2016), relevant decisions are often made at several levels not only by the top management. This research emergence has brought several scholars of the I/O domain to extend again decision research inside organizations at several roles.

### Linking Advances in Decision-making with the I/O Research Domain: the State of Art

The organizational research in decision-making is strictly connected to models used to diagnose the environment of study. Recent explorative studies have started to implement decision-making constructs within organizational models and the I/O variables related. For instance, the recent job demands-resources decision-making model (JD-R-DM, see Gordon et al., 2015), a version of the JD-R model (Demerouti et al., 2001), has proposed how the decision-making styles (Epstein et al., 1996) mediate contextual factors (i.e., job demands, job resources, work-engagement, job performance, etc.). In one of the confirmed hypothesis of the model, it shows the mediator role of the analytical decision-making in relation to job demands and in-role performance, in which: “*regulation of job demands is important to positively influence performance.*” (Gordon et al., 2015), which is also what we can expect from self-regulation mechanisms involved in decision-making considering the perspective of Bandura and Jourden (1991). On the other hand, the JD-R-DM model does not include an interaction between the analytical decision-making and job demands, but it is presumable that different levels of demands in relation to a decision-making construct such as the DMCy could affect job performance. This because of two reasons: first, DMCy has an impact on the self-regulation processes and then on the accomplishment of task targets (Byrnes et al., 1999; Byrnes, 2013); second, cognitive analytical style shows to be a convergence measure of the decisional competence (Finucane and Gullion, 2010). On the other hand, the decision-making styles assess the ways in which individuals approach decision-making, the competence instead measures how well individuals make decisions and it is usually considered as a construct trait-like^2^. For this reason, it is plausible that a competence such as the DMCy, instead of being a mediator of the process determined by the work environment (as for cognitive analytical style), is more likely to be a reliable antecedent of job performance by interacting with job demands.

Another consideration concerning the JD-R-DM model regards the absence of the classical JD-R exhaustion component, whereas work engagement and its relation with decision-making processes, for theoretical reasons concerning the role of positive affects in cognitive styles (Fredrickson, 2003), have been widely explored in the model. For evident methodological research limits, for what concerns new extensions of the JD-R model and in line with the research purpose, it is reasonable to consider only some components or relationships of the original model. In relation to the theoretical constructs so far presented, no study yet has analyzed the role of exhaustion on performance together with DMCy, DEM in the JD-R framework. To the best of our knowledge, we can assume that, if DMCy is related to task accomplishment (Byrnes et al., 1999; Byrnes, 2013), a relationship with in-role performance, actually defined as outcome of processes that directly serve organizational targets (Motowidlo and Van Scotter, 1994), is expected. Whereas, as seen, higher DEM in combination to a resourceful work environment should be positively related to the extra role performance. As well, in fact job resources are the most important predictors of extra-role performance, where job demands (if not particularly high) are associated to in-role performance (Bakker et al., 2004). For what concerns relationships with decision-making processes, we can presume that experienced exhaustion can alter choice processes by impairing the self-regulation mechanism of DMCy, which in turn can affect the performance in carrying out tasks (Humphreys and Revelle, 1984). Exhaustion also may drain perceived personal resources, leaving the executive function less capable of carrying out activities (Demerouti et al., 2001; Brotheridge and Grandey, 2002; Goldberg and Grandey, 2007). As well, the absence of some job resources has social consequences that can affect the good use of those decision competences related to the environment and ultimately working with others (Peterson et al., 2008). Job demands instead have been revealed to be an important regulator capable of influencing task performance (Gordon et al., 2015), probably in relation to a construct such as the DMCy.

**FIGURE 1:**
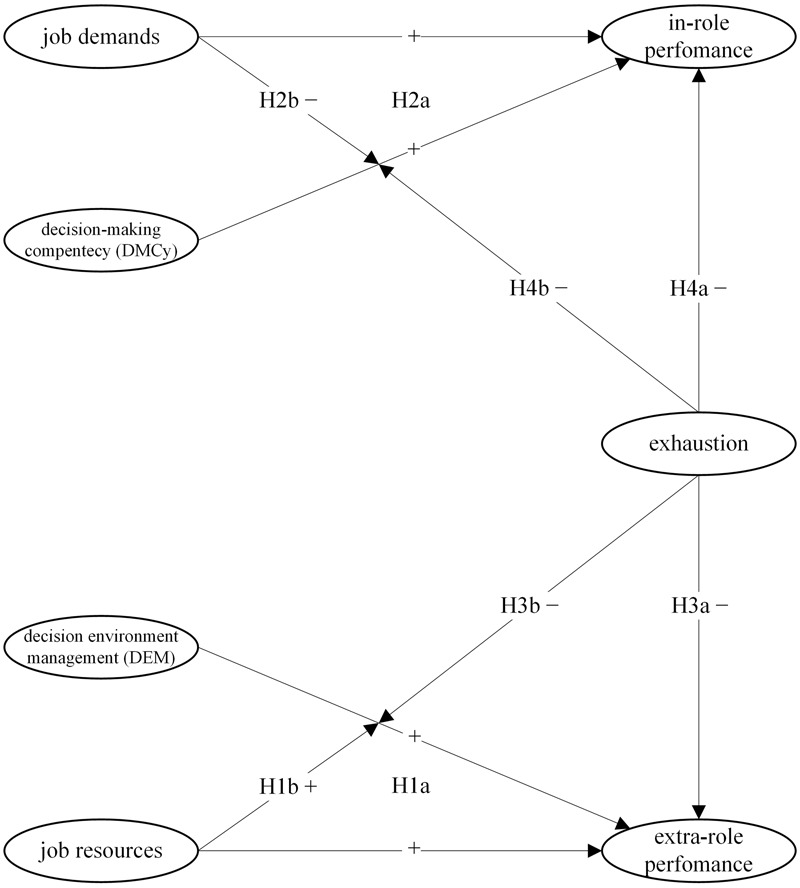
**Hypothesized Model 1 with interactions between decision competences and performance dimensions**.

Thus, we make the following hypotheses: Job resources and DEM are positively related to extra-role performance (H1a: DEM, job resources→extra-role performance). In addition, job resources moderate the effect of DEM on extra-role performance, so that extra role performance is positively influenced by the combination of high levels of job resources and DEM because such employees are able to better manage the presence of resources at work (H1b: DEM × job resources→extra-role performance). Job demands and DMCy are positively related to in-role performance (H2a: DMCy, job demands→in-role performance). Additionally, high levels of job demands can impair decision processes by negatively interacting with employees low in DMCy. Demands’ information overload is frequent in bad decision makers, resulting in scarce performance in carrying out their task targets (H2b: DMCy × job demands→in-role performance). Exhaustion is negatively associated with both kinds of performance (H3a: exhaustion→extra-role performance; H4a: exhaustion→in-role performance). Moreover, low levels of exhaustion have a positive moderation effect on the relationships between decision making competences (i.e., DEM, DMCy) and performance. Energetic individuals are usually successful in decision-making processes and report higher performance standards than their exhausted counterparts (H3b: DEM × exhaustion→extra-role performance; H4b: DMCy × exhaustion→in-role performance). A graphical representation of all the combined hypotheses mentioned is presented in Model 1 of **Figure 1**.

## Materials and Methods

The Ethical Review Committee at the University of Verona approved the study. We administered via mail 258 paper-and-pencil questionnaires to three Italian companies operating in the private service sector (i.e., administrative office sector, general service assistance, company support services). A total of 208 employees filled and returned the questionnaire (response rate 80%). The sample includes 132 females (63%). Their ages range between 20 and 60 years with an average of 41 years (*SD* = 9.65). The majority of the sample has higher general secondary education or vocational training (24%) or a high school degree (51%), 11% a bachelor degree and 7% a master degree. Most participants work as clerks (63%) or general workers (33%), only 4% are company managers or executives. Regarding supervision roles, 80% of the samples do not have supervising roles, 17.5% of the samples supervise between 1–5 persons, only five participants supervise more than five people.

### Instruments

*Decision-making Competency*: to assess this emergent construct, three components of the Decision-Making-Competency Inventory scale (DMCI) have been used (Miller and Byrnes, 2001). The DMCI scale has been created to assess some key aspects of decision-making skills by asking participants to report on their way of making decisions when they face important choices. Because we applied this measure to the organizational domain, each item began with the stem, “*When I have a big decision to make in the workplace…”* In relation to the present research, our interest focuses on the self-regulation process and consequently to the following three components: The person’s sense of self-determination in critically evaluating options (reversed scored, e.g., *“When I have a big decision to make about doing something that requires my skill, I often make a bad decision because I either underestimate or overestimate how good I am at something”)*, self-appraisal (reversed scored, e.g., *“I just go with a decision that all my colleagues are going with”)*, and the adequate self-confidence level in decisions (e.g., *“I usually believe that I will make a good decision”*). Respondents are asked to indicate on a five-point scale how much like them each statement is, with choices ranging from 1 (not at all like me) to 5 (very much like me).

#### Decision Environment Management (DEM)

To assess this construct, we used the Decision Environment Management Inventory (DEMI; Ceschi et al., submitted). The instrument is meant to ask participants to recall hypothetical decision scenarios usually present in the workplace (e.g., cost management choice, multiple job task situations, events organization, personnel relations, etc.), in which the quality of decisions can be differently affected or supported by the presence of some aspects of the work environment, such as the relation with the supervisor, with colleagues, the workload, some specific work activities, etc. Specifically, the instrument is composed of 17 items and assesses the three following components*: interpersonal behavioral determinants, properties of the organizational environment, cognitive and analytical aspects*. All items start with the following sentence: “*How the following interpersonal/ cognitive activities/ organizational and work aspects would affect or support your decision-making at work?* Examples of items are: “*relaxed relations between you and colleagues” “working overtime” “having to deal with some activities that need logical skills*” *“bureaucracy annoyances of your organization.”* The respondent has to first think about his∖her current job experience, and, by using the scale reported, answer the items. Because the items present positive or negative aspects which can affect the goodness of decision-making at work, the respondents has the possibility to answer them by using a seven point Likert scale, ranging from 1 = in a very bad way, to 7 = in a very good way. For more information regarding the scale, see the Appendix, in Supplementary Materials.

#### Job Resources

Three job resources were included in the questionnaire: feedback, opportunities for professional development, and social perceived support from colleagues. Feedback was measured with a three-item scale. Example items are “*I receive sufficient information about my work objectives*” and “*My job offers me opportunities to find out how well I do my work*” (1 = never, 5 = always). Opportunities for professional development were measured with the three-item scale of Bakker et al. (2003), including: “*My work offers me the opportunity to learn new things*” and “*I have sufficient possibilities to develop myself at work*” (1 = totally disagree, 5 = totally agree). Social support was measured with three items from the scale developed by Van Veldhoven and Meijman (1994). Example items are “*Can you ask your colleagues for help if necessary*?” and “*Can you count on your colleagues when you face difficulties at work?*” (1 = never, 5 = always).

#### Job Demands

Three job demands were included in the questionnaire: cognitive demands, emotional demands, and hassles. Cognitive demands were evaluated with a four-item scale (Bakker et al., 2003). A typical item of this scale is “*Does your work demand enhanced care or precision*?” (from 1 = never to 5 = always). Emotional demands were based on a scale developed by Van Veldhoven and Meijman (1994) and included four items. An example is “*Does your work put you in emotional situations*?” (1 = never, 5 = always). The Hassles scale (Bakker, 2014) was used to detect the level of perceived administrative hassles. It is composed of six items. Examples are: “*I have to deal with administrative hassles”; “I have many hassles to go through to get projects/assignments done”;* (1 = never, 5 = always).

#### Exhaustion

Three exhaustion items of the Oldenburg Burnout Inventory (Demerouti and Bakker, 2008) were used. Example items are “*There are days when I feel tired before I arrive at work*” and “*After my work, I usually feel worn out and weary*” (1 = totally disagree, 4 = totally agree).

#### Performance

Two types of job performance were assessed: in-role and extra-role performance. In-role performance relates to officially needed outcomes and behaviors that straight serve the organization aims (Borman and Motowidlo, 1997). Other labels sometimes used are job-specific task proficiency or simply task performance (Koopmans et al., 2012). General in-role performance was measured with three items, an example is: “*I achieve the objectives of my job*” (0 = Not at all characteristic, 6 = Totally characteristic). Extra-role performance (i.e., contextual performance) is defined as work behaviors and activities that are not necessarily related to work tasks but that contribute to the social and psychological aspects of the organization (Borman and Motowidlo, 1993). It was measured with other three items, such as: *“I managed to plan my work so that it was done on time”* (same previous respond scale). Both scales were derived from the questionnaire by Goodman and Svyantek (1999). In addition, a second instrument: the Individual Work Performance Questionnaire (IWPQ, Koopmans et al., 2012) was used to measure the two types of performance. It is structured in three dimensions: the task performance (i.e., in-role performance), the contextual performance (i.e., extra performance) and the counterproductive work behavior (not considered in the present study). Compared to the previous scale, the questionnaire is composed of more items: five for the task performance, seven for contextual performance; the rate is expressed on a five-point rating scale (0 = never, 4 = very often). A task performance example item is: *“I managed to plan my work so that it was done on time,”* whereas a contextual performance one is: “*I actively participated in work meeting.*”

## Results

### Descriptive Statistics

**Table 1** shows the means, standard deviations, correlations, and the internal consistency indexes of the scales. All scales present acceptable reliability indexes. Both decision making measures showed not to be correlated, confirming an interdependence in measuring two distinct decision-making competences. In relation to socio-demographic variables, DMCy shows positive correlations with length in service and job position (*r* = 0.21, *p* < 0.01); DEM presents a positive correlation with the number of supervised staff (*r* = 0.20, *p* < 0.01). Consistent with our hypotheses, we found positive correlations between DMCy and in-role performance, and between DEM scores and extra-role performance on the other hand, partially confirming hypotheses H1a and H2a. DEM in addition shows a positive correlation with some job resources (feedback = 0.19, *p* < 0.05; professional development = 0.33, *p* < 0.01), instead no relationship between DMCy and job demands emerged. Both decision-making measures revealed negative correlations with exhaustion (DEM = -0.20, *p* < 0.05; DMCy = -0.27, *p* < 0.01) partially confirming H3a and H4a. Next analyses will test the direction of such relationships in order to fully confirm hypotheses.

**Table 1:** Means, standard deviations (SD), internal consistencies (on the diagonal) and correlations among socio-demographics and study’s variables.

		*M* (*SD*)	1	2	3	4	5	6	7	8	9	10	11	12	13	14	15	16	17	18	19
1.	Gender	0.37 (0.48)	–																		
2.	Age	40.73 (9.65)	-0.18*	–																	
3.	Education	3.25 (1.36)	0.06	-0.12	–																
4.	Length in service	10.70 (7.12)	0.11	0.42**	-0.14	–															
5.	Number of staff supervised	1.56 (1.28)	-0.14*	0.06	0.26**	0.00	–														
6.	Job position	1.75 (0.59)	0.04	0.03	0.36**	0.25**	0.23*	–													
7.	Cognitive demands	3.67 (0.87)	-0.15*	-0.06	0.13	-0.03	0.16*	0.21**	(0.87)												
8.	Emotional demands	2.42 (0.86)	-0.27**	0.07	0.18**	-0.15*	0.17*	0.14*	0.41**	(0.84)											
9.	Hassle	2.62 (0.85)	0.11	-0.20**	0.17*	0.10	0.30**	0.11	0.15*	0.02	(0.85)										
10.	Social support	3.24 (0.84)	0.04	-0.21**	-0.05	-0.15*	-0.03	0.00	0.19**	0.15*	-0.09	(0.71)									
11.	Feedback	2.86 (0.82)	0.01	-0.01	0.00	-0.21**	0.08	-0.04	0.23**	0.12	-0.04	0.29**	(0.80)								
12.	Professional development	3.40 (0.80)	0.00	0.02	0.12	-0.01	0.25**	0.07	0.23**	0.01	0.17*	0.26**	0.47**	(0.85)							
13.	Exhaustion	2.39 (0.58)	-0.03	-0.21**	-0.12	-0.09	-0.03	-0.14*	-0.13	0.17*	0.11	0.02	-0.04	-0.18**	(0.75)						
14.	Decision-making competency (DMCy)	2.15 (0.44)	0.03	0.11	0.04	0.19**	0.02	0.21**	0.03	-0.08	0.02	-0.03	-0.04	-0.09	-0.27**	(0.79)					
15.	Decision environment management (DEM)	2.65 (0.60)	0.07	-0.01	0.17*	0.08	0.20**	0.10	0.14*	-0.05	0.14*	0.10	0.19**	0.33**	-0.20*	0.03	(0.82)				
16.	General in-role performance	3.21 (1.05)	-0.03	0.12	0.00	0.00	0.03	0.18**	0.12	0.02	-0.02	0.06	0.18*	0.09	-0.24**	0.33**	0.17*	(0.85)			
17.	General extra-role performance	3.21 (1.23)	0.11	-0.18*	0.10	0.14	0.18*	0.14*	0.05	-0.07	0.24**	0.06	-0.01	0.16*	-0.06	0.09	0.34**	0.29**	(0.82)		
18.	Task performance	2.66 (0.68)	0.01	0.08	0.00	0.00	0.03	0.09	0.28**	0.13	-0.09	0.10	-0.16*	0.09	-0.20**	0.20*	0.24**	0.49**	0.18*	(0.82)	
19.	Contextual performance	1.85 (0.86)	-0.06	-0.08	0.24**	-0.17*	0.41**	0.18**	0.34**	0.17*	0.25**	0.10	0.35**	0.37**	-0.13	0.04	0.24**	0.17*	0.22**	0.22**	(0.84)

*Note. Gender: 0 = woman; 1 = men; Education: 1 = Elementary school; 2 = Lower general secondary education; 3 = Higher general secondary education; 4 = Preparatory vocational education; 5 = Higher professional education; 6 = Bachelors’ degree; 7 = Masters’ degree; 8 = Ph.D. Length of service: Tenure expressed in years; Employees managed: 1 = Up to 2 supervised co-workers; 2 = 3 to 5 supervised co-workers; 3 = 6 to 10 supervised co-workers; 4 = 11 to 25 supervised co-workers; 5 = More than 25 supervised co-workers; Job position: 1 = Worker; 2 = Senior clerk; 3 = Manager; 4 = Executive; ^∗^*p* < 0.05; ^∗∗^*p* < 0.01.*

### Structural Equation Modeling (SEM) Testing

Given the intercorrelations of job demands, job resources and the two performance dimensions, standardized composite scores were computed prior to hypotheses and model testing (see Model 1 in **Figure 1**). All variables including the moderation terms have been patterned as latent factors with a single indicator. All latent factors were adjusted for random measurement error by establishing the random error variance of each construct corresponding to the product of its variance and the quantity minus its original internal consistency. Variables that considered moderator effects were constrained in accordance with Cortina et al. (2001), and standardized in order to estimate the reliability of the interaction terms. Such procedure is based on the original reliability of both variables used to form a product term and the correlation amongst the two latent variables as value for the path from the latent interaction factor to its indicator. As for all model variables, the error variance of the indicator of the latent interaction factor was set equal to the product of its variance minus its reliability. Finally, for DMCy, DEM, job demands, job resources, exhaustion, and two performance dimensions, the path from the latent variables to their corresponding observed variable was equal to the square root of reliability of the observed score. In testing the hypothetical Model 1 with all the interactions considered and performed with the maximum likelihood estimation method, fit indices suggested an acceptable model [x^2^(36.31, *df* 25, *p* > 0.06); GFI = 0.97; RMSEA = 0.047; CFI = 0.94] (**Table 2**).

**Table 2:** Goodness-of-Fit Indices (Maximum-Likelihood Estimates) for the Structural Equation Models proposed.

		*x*^2^	*df*	*p*	GFI	RMSEA	CFI
1.	Model 1	36.31	25	0.06	0.97	0.047	0.94
2.	Model 2	27.41	17	0.05	0.98	0.054	0.95

*GFI, goodness of fit index; RMSEA, root mean square error of approximation; CFI, comparative fit index.*

Consistent with our hypotheses, most of the main and moderation effects have been found significant and in the expected direction. Hypotheses 3 and 4 have been just partially confirmed: the relationship between exhaustion and extra-role performance (H3a) has been found not significant in the model, together with the expected interaction between DMCy and exhaustion toward in-role performance (H4b). Therefore, a second model (Model 2) was tested without the moderating effect of exhaustion in the DMCy in-role performance relationship. The elimination of the interaction path resulted in an increment to an acceptable CFI value (Hu and Bentler, 1999) and in an acceptable small increment of RMSEA value [x^2^(27.41, *df* 17, *p* > 0.05); GFI = 0.98; RMSEA = 0.054; CFI = 0.95].

**FIGURE 2:**
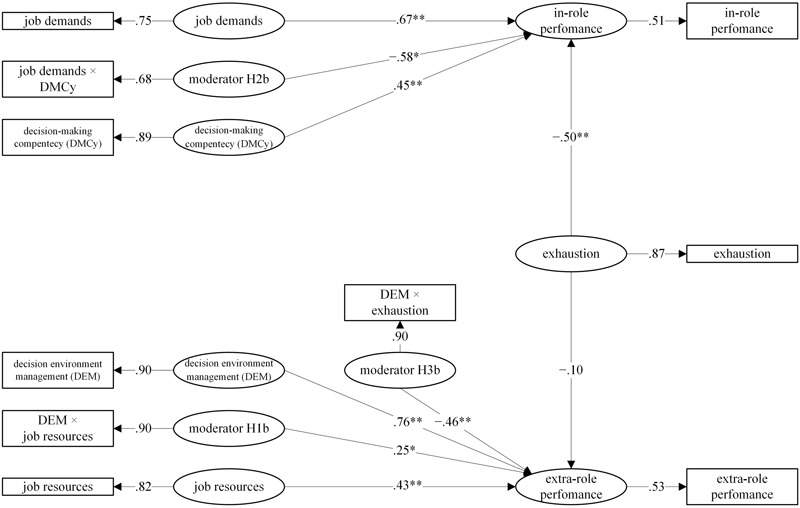
**Tested Model 2 with interactions between decision competences and performance dimensions (^∗^*p* < 0.05; ^∗∗^*p* < 0.01)**.

Model 2 showed the same significant relations compared to model 1 in terms of main effects. Decision Making Competency (β = 0.45, *p* < 0.01), job demands (β = 0.67, *p* < 0.01) and exhaustion (β = -0.50, *p* < 0.01) were significantly related to in-role performance confirming H2a and H4a. Confirming hypothesis H1a, DEM (β = 0.76, *p* < 0.01) and job resources (β = 0.43, *p* < 0.01) were significantly related to extra-role performance. In addition, the structural equation modeling (SEM) confirmed three supposed interactions out of four involving different performance dimensions, as stated in hypotheses H1b (DEM × job resources → extra-role performance: β = 0.25, *p* < 0.05), H2b (DMCy × job demands → in-role performance: β = -0.58, *p* < 0.01) and H3b (DEM × exhaustion → extra-role performance: β = -0.46, *p* < 0.01). All the resulting relationships of Model 2 are graphically displayed in **Figure 2**.

### Simple Slope Analyses

**FIGURE 3:**
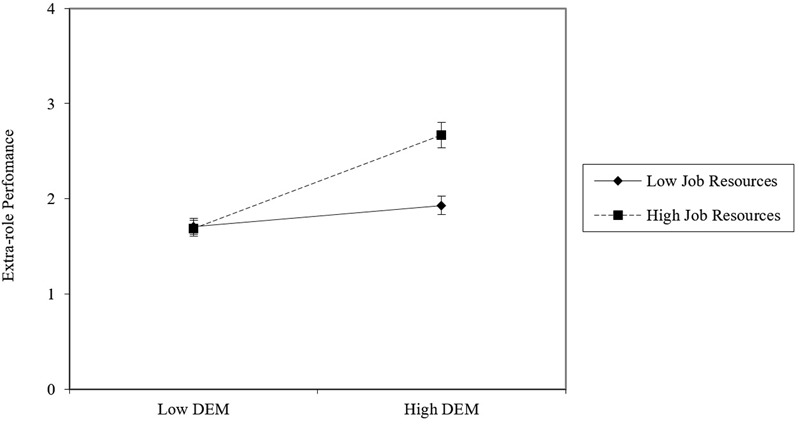
**Relationship between job resources and extra-role performance for low versus high decision environment management (DEM) employees**.

Simple slope analyses were performed to explore interactions emerged in the SEM. In relation to DEM, job resources and estimated H1b moderator (DEM × job resources → extra-role performance), results showed that for higher levels of job resources (+1 SD above the mean) there was a stronger positive relationship between DEM and extra-role performance (*B* = 0.425, *t* = 3.96, *p* < 0.001), whereas this relationships was less strong for lower levels of job resources (-1 SD below the mean: *B* = 0.180, *t* = 2.03, *p* < 0.05). A graphical representation of the moderation effect is presented in **Figure 3**, which shows how participants high in DEM benefit more from job resources when these are high, eventually resulting in higher extra-role performance.

**FIGURE 4:**
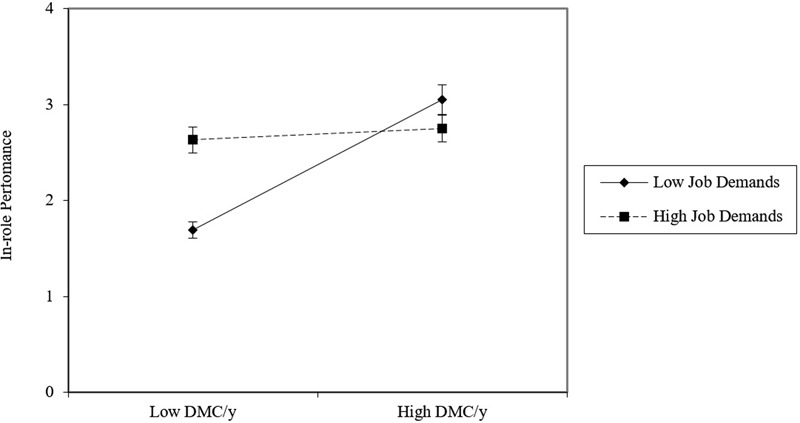
**Relationship between job demands and in-role performance for low versus high decision-making competency (DMCy) employees**.

Considering moderation related to hypothesis H2b (DMCy × job demands → in-role performance), the simple slope revealed that for low levels of job demands, DMCy is strongly and positively related to in-role performance (-1 SD under the mean: *B* = 0.551, *t* = 4.06, *p* < 0.001) and it becomes non-significant for higher levels of them (+1 SD above the mean: *B* = 0.184, *t* = 1.11, *p* = 0.268). **Figure 4** shows that high levels of in-role performance of participants high in DMCy do not coincide with in-role performance levels of subject low in DMCy when job demands are low.

**FIGURE 5:**
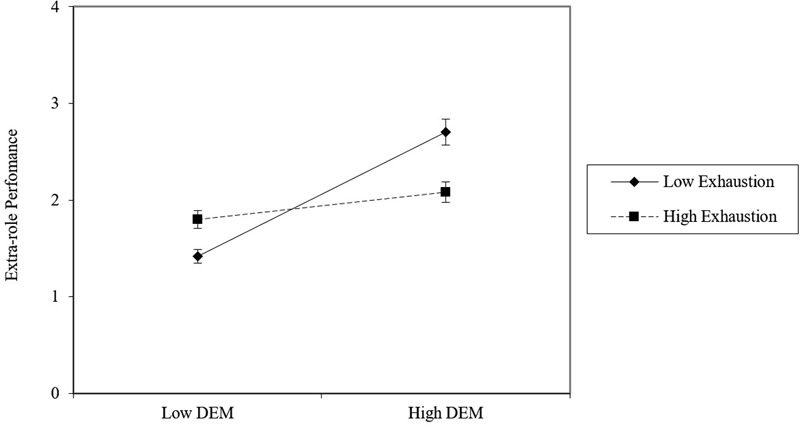
**Relationship between exhaustion and extra-role performance for low versus high decision environment management (DEM) employees**.

A graphical representation of the moderation effect of Hypothesis H3b (DEM × exhaustion → extra-role performance) is presented in **Figure 5**. It shows that extra-role performance is higher when DEM is high (versus low) for low exhaustion condition, whereas for subjects low in DEM the trend inverts. The interaction effect is significant for all the levels of the moderator (+∖- 1 SD), but, contrary to the first moderation pattern, the interaction effect follows a decremental trend: For low levels of exhaustion (-1 SD below the mean), DEM has a stronger positive relationship with extra-role performance, (*B* = 0.538, *t* = 5.11, *p* < 0.001), whereas the strength of this relationship is halved for higher levels of exhaustion (+1 SD above the mean: *B* = 0.246, *t* = 2.47, *p* < 0.05).

## Discussion

The present research links advances in decision-making and in organizational psychology, by presenting how self-regulation processes of decisions are related to performance, and how such relationship can be affected by the presence of exhaustion, job demands, and job resources. Novelties present in the current study are several. First, the methodological aspects here considered present a solution for an antithetical issue which sees decision-making studies mostly experimental and between-subject conceived, and I/O research based on psychometric instruments (Dalal et al., 2010). Newborn approaches in decision-making originated from studies of self-regulation competency dimension (Byrnes et al., 1999) and of environmental decision-making model by Bandura and Wood (1989) allow to overcome to this limit by using psychometric instruments developed on purpose for measuring decision-making competences at work. Second, the study extends decision making research applied to the I/O domain in the framework of the JD-R model. The model here proposed explores the role of the exhaustion component, decision-making competences (i.e., DMCy and DEM) and their interactions which have not been tested in the recent decision-making JD-R extension (Gordon et al., 2015). Third, we have extended studies on decision-making applied to the workplace, frequently confined to some privileged subjects, such as managers or specific professionals, to general workers. This in light of a research emergence which has brought several scholars to reconsider the new role assumed by the work environment and its effect on decision-making processes. Results of the present study reveal the importance of the sensitivity to environmental aspects in decision-making (DEM) as well the role of the competence of making good choices at work (DMCy) in relation to organizational variables. The importance of these characteristics illuminates how decision-making processes in the workplace improve different job performance types and how some JD-R variables can positively or negatively affect such processes.

### Decision-making Competency (DMCy) and Decision Environment Management (DEM) at Work

This study confirms that in-role performance depends on DMCy as well as job demand levels. In addition, for low levels of job demands, DMCy is strongly and positively related to in-role performance, whereas such interaction disappears for high levels of them. Although research has robustly shown that high chronic job demands and exhaustion adversely affect performance (Demerouti et al., 2001; Bakker et al., 2003, 2004) very few research has focused on its possible effects on decision-making in relation to low job demand levels. A possible explanation comes from the definition of DMCy, in which the self-regulation of people allows an efficiently feeling of control over processes of adaptation to the work environment (Miller and Byrnes, 2001). The self-regulation processes for people with high DMCy could allow the development of strategies able to overcome to frustrations due to changes in job demands. Additionally, it is important to mention that job demands need not to be necessarily negative (if not particularly high), and they still are an indirect index of productivity and performance related to the task (Bakker and Demerouti, 2007). For example, after crisis periods which some Italian companies in the private service sector are now facing, employees with high DMCy can better adapt to new job conditions by developing strategies (e.g., finding new clients, developing international networks, restructuring the organization, introducing new technologies) to overcome environmental conditions and maintaining high performance. This is in line with the DMCy definition which sees a good decision maker as able to promote the implementation of strategies to establish a sense of control to adapt to unpredictable environments (Miller and Byrnes, 2001).

On the other hand, the DEM is conceptualized as the sensitivity toward environmental aspects relevant for decision processes, which, in turn, may be associated with extra-role performance. As seen, the impact of these environment variables on decision-making processes is relevant and already known, especially in relation to job resources (De Dreu and Weingart, 2003; Kozlowski and Ilgen, 2006; Ceschi et al., 2014). Our study suggests that job resources may especially affect the performance of individuals who are more receptive to environmental conditions which results in an advantage for them. In complex decision-making environments, high DEM decision makers develop better composite rules, making it effortlessly to assess the source of multiply produced effects, and making effective use of insightful outcome feedbacks (Wood and Bandura, 1989; Bandura, 1997). When carrying out these activities, employees have to cope with plenty of limitations and drawbacks that frequently perturb self-evaluative repercussions impairing decision-making processes, this especially if they are low in DEM. For the same reason, individuals who reported higher levels of DEM also report better with extra-role performance in low exhaustion condition, when their energetic status dispose of the enough cognitive resources for making good decisions.

### Two Different Processes, One Common Regulation System?

Relations found among these characteristics suggest the presence of two different constructs related to decision-making processes. It is interesting the fact that both of them show the presence of an insensitivity to moderators, but in an opposite way: where subjects with low levels of DMCy are sensitive to demands, high DEM levels seem to increase the sensitivity toward resources. DMCy seems to be a construct oriented to performance protection versus job demands, activating the regulation at the increasing of subjective effort (Demerouti et al., 2014). On the other side, DEM enhances the sensitivity toward resources in relation to extra-role performance and probably in relation to the motivational process proposed by the JD-R model (Bakker et al., 2003). The motivational process assumes that job resources have motivational potential and lead to high work engagement and extra-role performance, limiting the development of job strain (Bakker et al., 2005). This could explain why employees with high level of DEM perform better in absence of harsh work conditions, because they are more inclined to be negatively affected by exhaustion. On the other hand, only future research could confirm such result interpretation, because the present research lacking of the work engagement indicator did not allow to test such mediated relation.

Considering differences between the two decision-making constructs, a process that can explain their impact on performance relies on the self-regulatory mechanism. As seen, self-regulatory mechanisms have considerable impact on how well cognitive-processing systems work (Wood et al., 1990). The conception of ability with which employees approach complex activities are likely to have a significant impact on the self-regulatory influences that govern ongoing motivation and personal accomplishments in complex decision-making environments; which is also consistent with the definition of self-regulation of the DMC∖y approach (Byrnes et al., 1999). Self-regulation is based on generating, evaluating, selecting, and learning from goal-directed choices while simultaneously managing uncertainty, complications, time pressure, that may otherwise interfere with the goals attainment.

### DMCy and DEM as Detectors of Individual Differences among Organizational/Work Variables

Some considerations need to be reported in relation to differences among participants and the relations found with DMCy and DEM. Consistent with literature on individual differences, where people with low decision-making competence are related to greater risk-taking, interpersonal strengths and difficulties, and high levels are considered as predictors of such real-world success (Parker and Fischhoff, 2005; Weller et al., 2012, 2015), DMCy is positively related to job position. Managerial roles are often associated with good decision-making ability, mostly analytical, as evidence has showed (Dane and Pratt, 2007). DMCy is also related to work experience. In the workplace, experienced workers reliance on less cognitively demanding strategies would possibly not always be a disadvantage, as these more straightforward strategies may lead to adaptive behaviors as a result of an equilibrium between individual potential and the demands of a job condition (Mata et al., 2007, 2009, 2011). The study has provided significant results related to the decision-making literature.

Another significant relation detected is between DEM and the number of supervised staff, where individuals which present such higher sensitivity to resources in decision-making are also in position of governing more personnel. This is in line with the theoretical definition of DEM which is expected to interact positively with job-resources such as the supervised staff, to accomplish performance targets (Wood et al., 1990). As for job resources like feedbacks or social rewards, the strength of these variables is intrinsically related to the sensitivity toward them and subsequently on decision-making processes which can positively impact on employee’s co-working. Therefore, negative environments can be responsible for exhaustion and work disengagement especially in those individuals sensitive to the choice regulation processes dependent on work context.

### Limitations and Future Research

Limitations of this study can be considered basically four. First, in common with several I/O studies, the present research lacks an objective measure of performance (Spector, 2006). In part, this lack has been balanced with the use of more scales to measure the in-role and the extra-role performance, such as the IWPQ (Koopmans et al., 2012) and the performance scale present in the JD-R questionnaire by Bakker (2014). A second limitation is due to the absence of a second self-regulation/dysregulation measures. Partially this limit is overcome by the use of a convergent measure based on the three self-regulation scales of the DMCI (Miller and Byrnes, 2001) which has been developed starting from the “*self-regulation decision-making model”* (Bandura and Wood, 1989; Byrnes, 2013) with the intention of assessing self-regulation in making choices. The third limitation of the study belongs to the decision-making competence construct (Bruine de Bruin et al., 2012), which differs from the competency one because of the use of heuristics and biases tasks to test the decisional ability (Miller and Byrnes, 2001). Future research considering the use of heuristics and biases tasks could bring more evidence able to explain such dynamics. The last limitation regards the cross-sectional design of the present study, which does not allow to observe causality of the relationships between predictors and outcomes by controlling for stabilities. Future studies should examine such relationships over time, in relation to training program for instance. Considering the perspective assumption that is actually considering the decision-making competence as a construct like-trait (Finucane and Gullion, 2010; Bruine de Bruin et al., 2012), scholars should also investigate whether specific training programs (i.e., de-biasing) could possibly improve the de-biased decisions. We suggest the development of longitudinal de-biasing programs (Fischoff, 1982; Larrick, 2004; Gerling, 2009; Soll et al., 2014) combined to job crafting interventions (based on JD-R framework; Tims and Bakker, 2010; Bakker et al., 2012; Petrou et al., 2012; Tims et al., 2012; Demerouti and Bakker, 2014) especially in relation to a qualitative measure based on diaries studies at work, to test decision-making and performance improvements. In relation to DEM, training courses could develop the awareness, the intrinsic management, and the self-regulation in relation to organizational variables and decision-making processes. Training could improve collaborative processes, such as shared decision-making, they would allow the improvement of the management of decisional environments, which in turn will positively increase performance and permit to better deal with environment exhaustion. Ultimately, considering the effects of employee well-being on decision-making research will consider more in the future the psychosocial effects of individuals at work, despite of just focusing on the company performance as the only decision outcome.

## Author Contributions

All authors listed, have made substantial, direct, and intellectual contribution to the work, and approved it for publication.

## Conflict of Interest Statement

The authors declare that the research was conducted in the absence of any commercial or financial relationships that could be construed as a potential conflict of interest.

## Footnotes

1 Decision-Making Competence (Parker and Fischhoff, 2005) is an objective measure of the competence which assesses whether individuals reach satisfactory outcomes in decision-making. Questions are based on seven assorted tasks groups for a combined total of 87 items. The decision-making competency measure (Miller and Byrnes, 2001) instead is based on a self-report instrument to assess self-regulation and the use of metacognitive processes to examine choice options and master decisions.

2 For more information concerning instruments used to asses styles and competence in decision-making we invite to see the decision making individual differences inventory section hosted in the website of the Society for Judgment and Decision Making (www.sjdm.org).
